# Supplementary material for: Knockdown of a Novel Gene OsTBP2.2 Increases Sensitivity to Drought Stress in Rice
Source: Genes (Basel). 2020 Jun 8;11(6):629. doi: 10.3390/genes11060629 (PMC7349065; doi:10.3390/genes11060629)
Supplement: Supplementary file 1 [file genes-11-00629-s001.zip › Supplementary Files/Figure S#.pptx]

## Slide 1
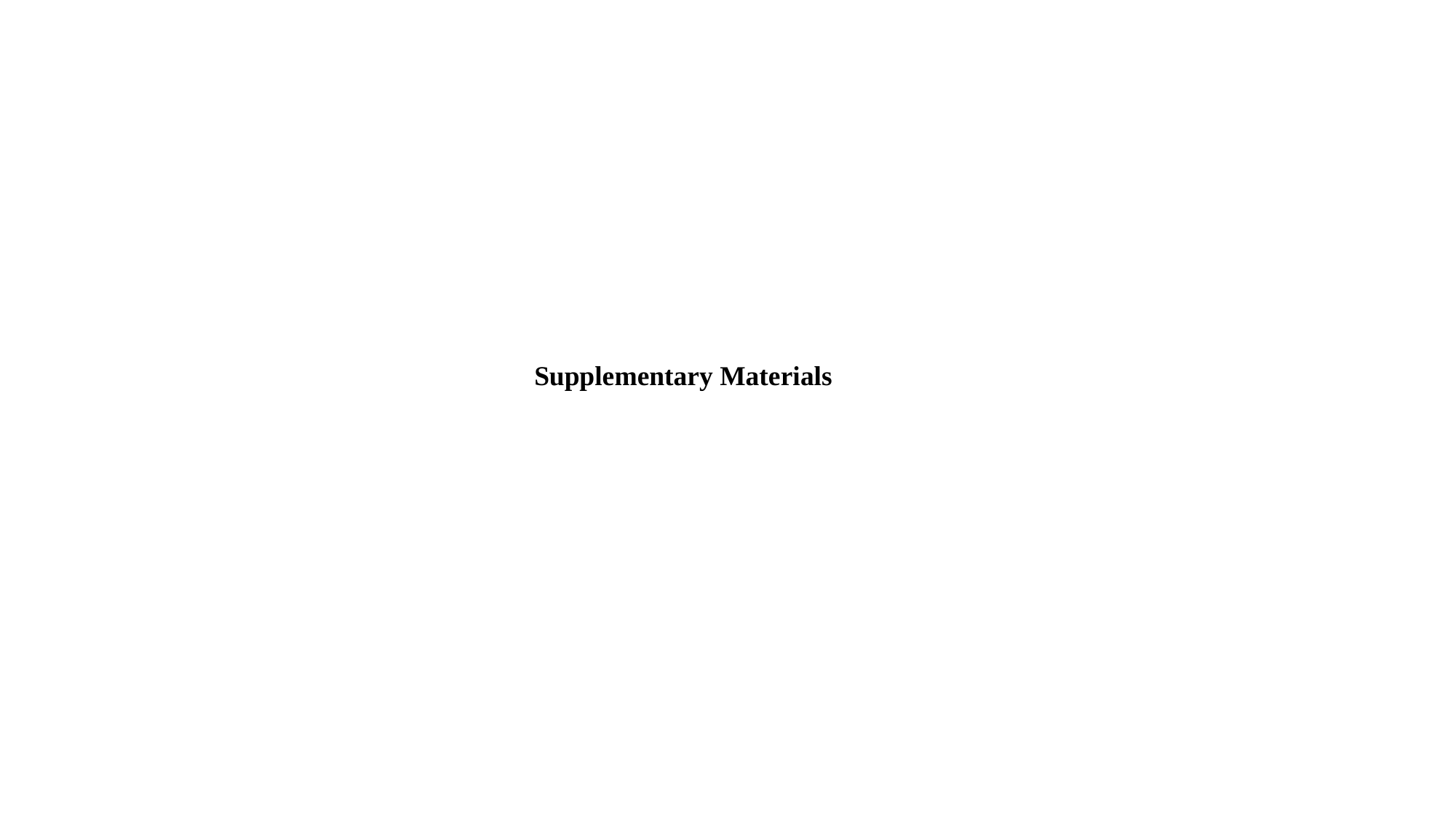

Supplementary Materials

## Slide 2
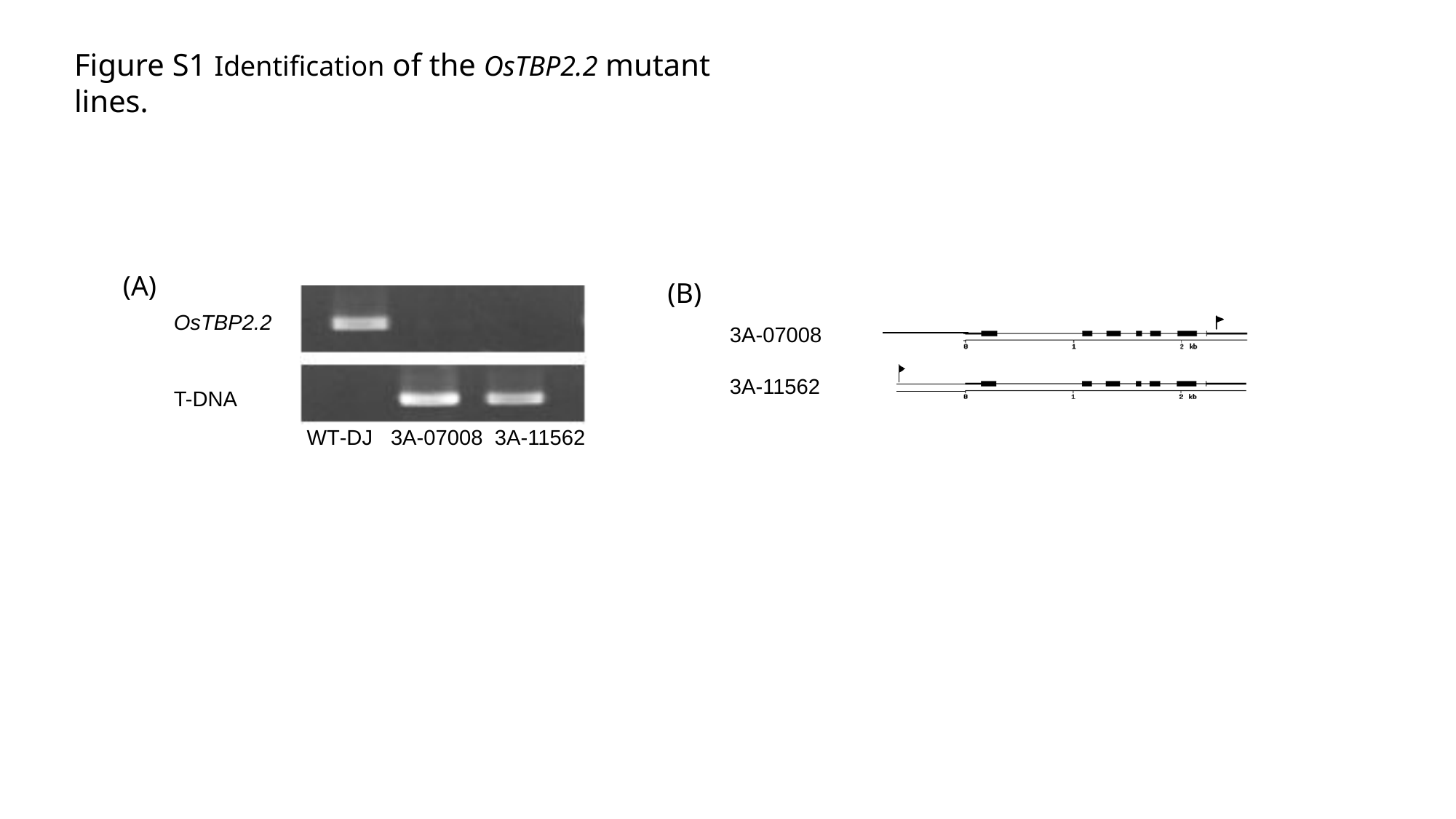

Figure S1 Identification of the OsTBP2.2 mutant lines.
(A)
(B)
OsTBP2.2
T-DNA
 WT-DJ 3A-07008 3A-11562
3A-07008
3A-11562

## Slide 3
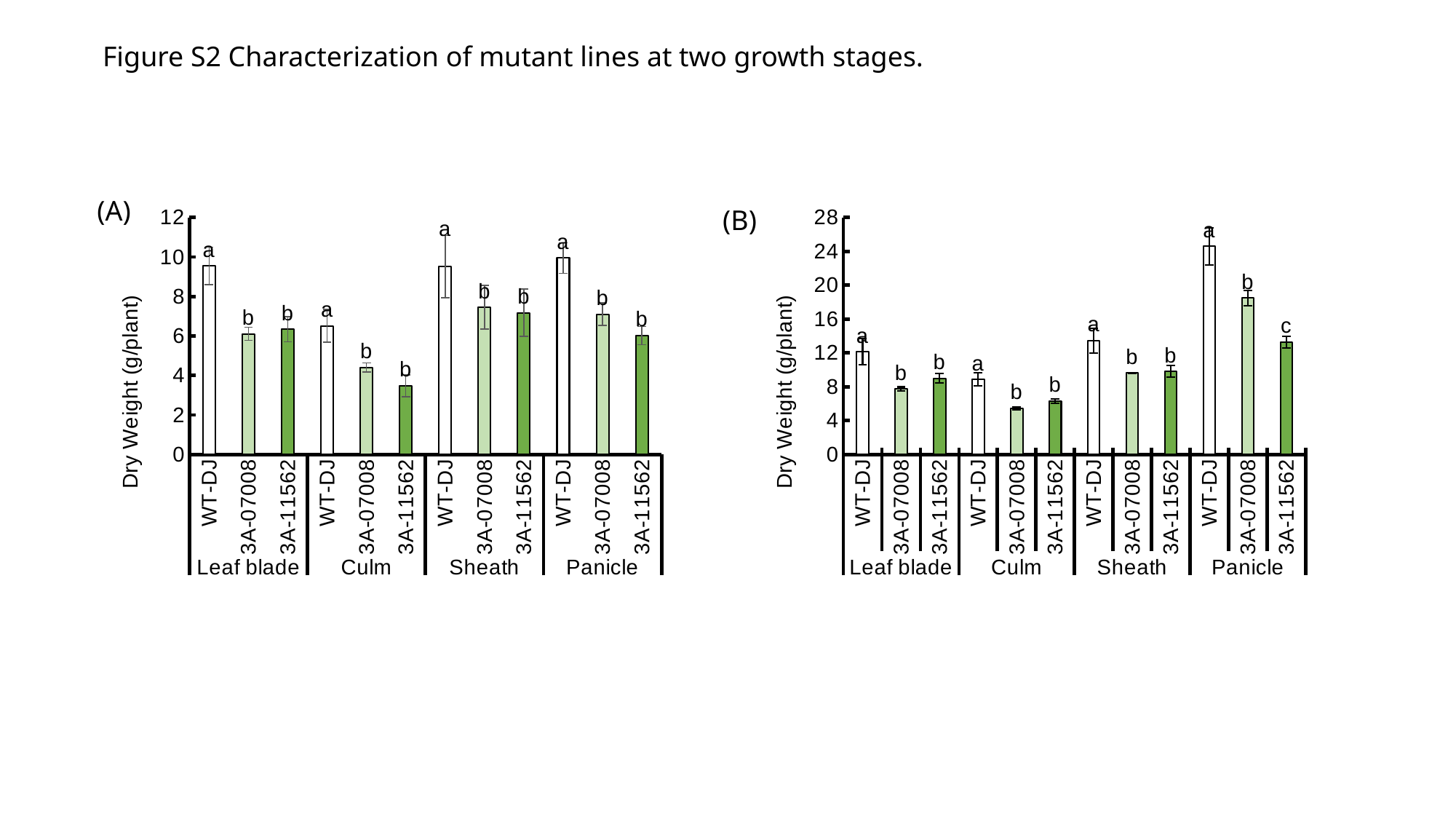

Figure S2 Characterization of mutant lines at two growth stages.
(A)
### Chart
| Category | |
|---|---|
| WT-DJ | 9.551 |
| 3A-07008 | 6.1056 |
| 3A-11562 | 6.33625 |
| WT-DJ | 6.5065 |
| 3A-07008 | 4.4012 |
| 3A-11562 | 3.4704000000000006 |
| WT-DJ | 9.506333333333332 |
| 3A-07008 | 7.4535 |
| 3A-11562 | 7.171666666666667 |
| WT-DJ | 9.94425 |
| 3A-07008 | 7.098750000000001 |
| 3A-11562 | 6.01625 |(B)
### Chart
| Category | |
|---|---|
| WT-DJ | 12.13 |
| 3A-07008 | 7.737 |
| 3A-11562 | 8.996666666666668 |
| WT-DJ | 8.858666666666666 |
| 3A-07008 | 5.436249999999999 |
| 3A-11562 | 6.303333333333335 |
| WT-DJ | 13.440333333333333 |
| 3A-07008 | 9.622 |
| 3A-11562 | 9.805 |
| WT-DJ | 24.59825 |
| 3A-07008 | 18.49725 |
| 3A-11562 | 13.2805 |

## Slide 4
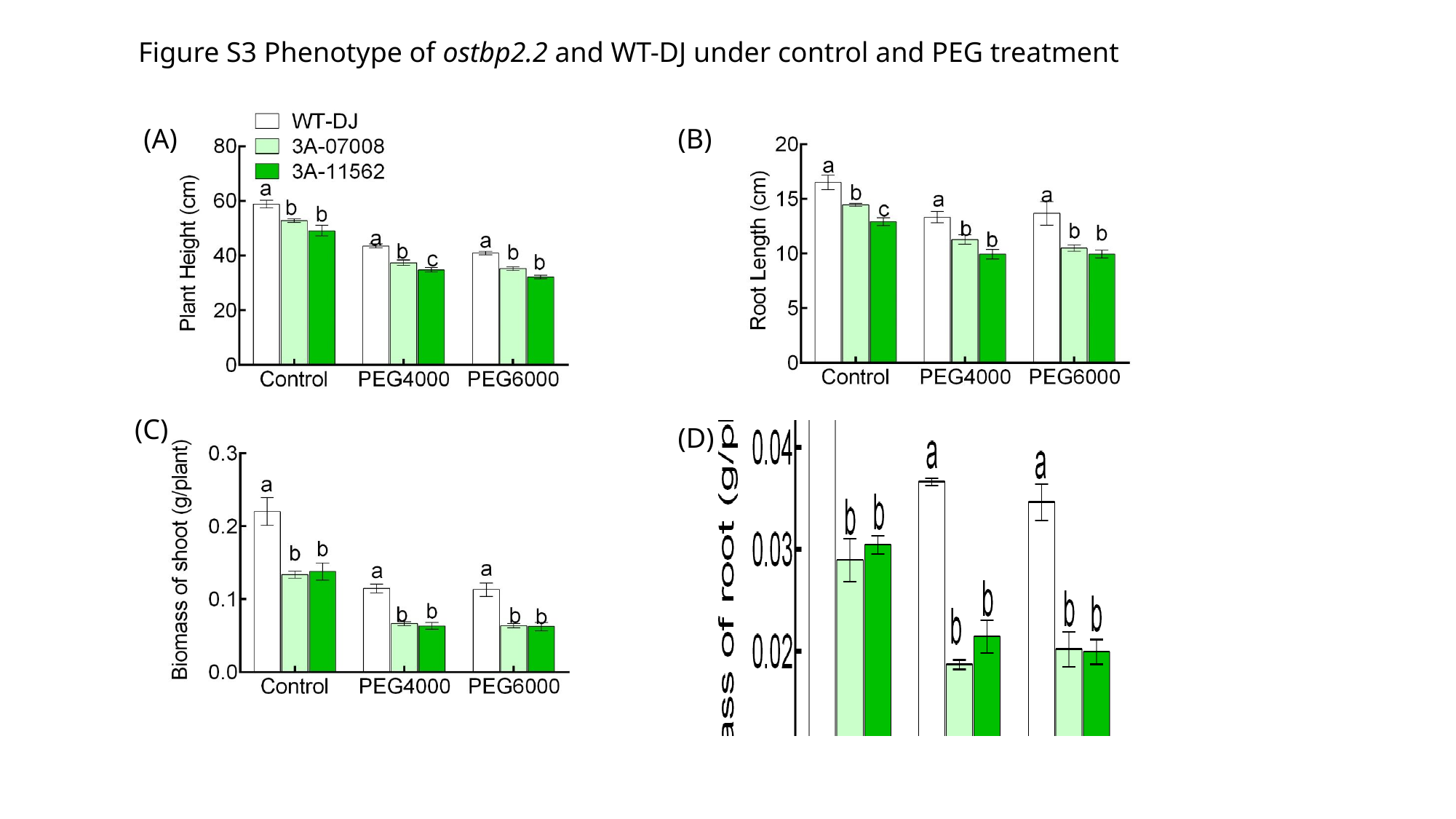

Figure S3 Phenotype of ostbp2.2 and WT-DJ under control and PEG treatment
(A)
(B)
(C)
(D)

## Slide 5
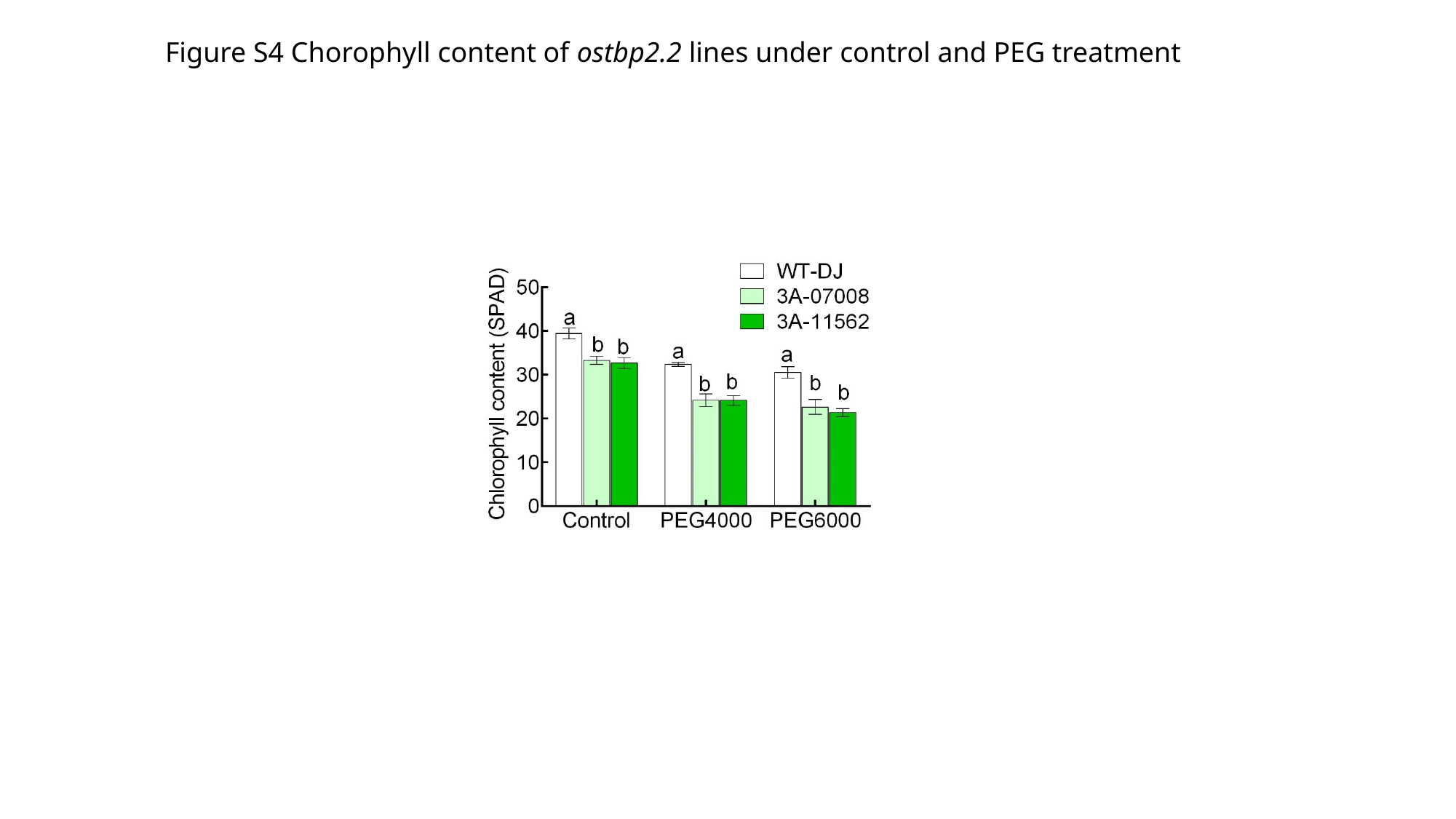

Figure S4 Chorophyll content of ostbp2.2 lines under control and PEG treatment
