# Supplementary material for: Knockdown of a Novel Gene OsTBP2.2 Increases Sensitivity to Drought Stress in Rice
Source: Genes (Basel). 2020 Jun 8;11(6):629. doi: 10.3390/genes11060629 (PMC7349065; doi:10.3390/genes11060629)
Supplement: Supplementary file 1 [file genes-11-00629-s001.zip › Supplementary Files/Table S1 .pptx]

## Slide 1
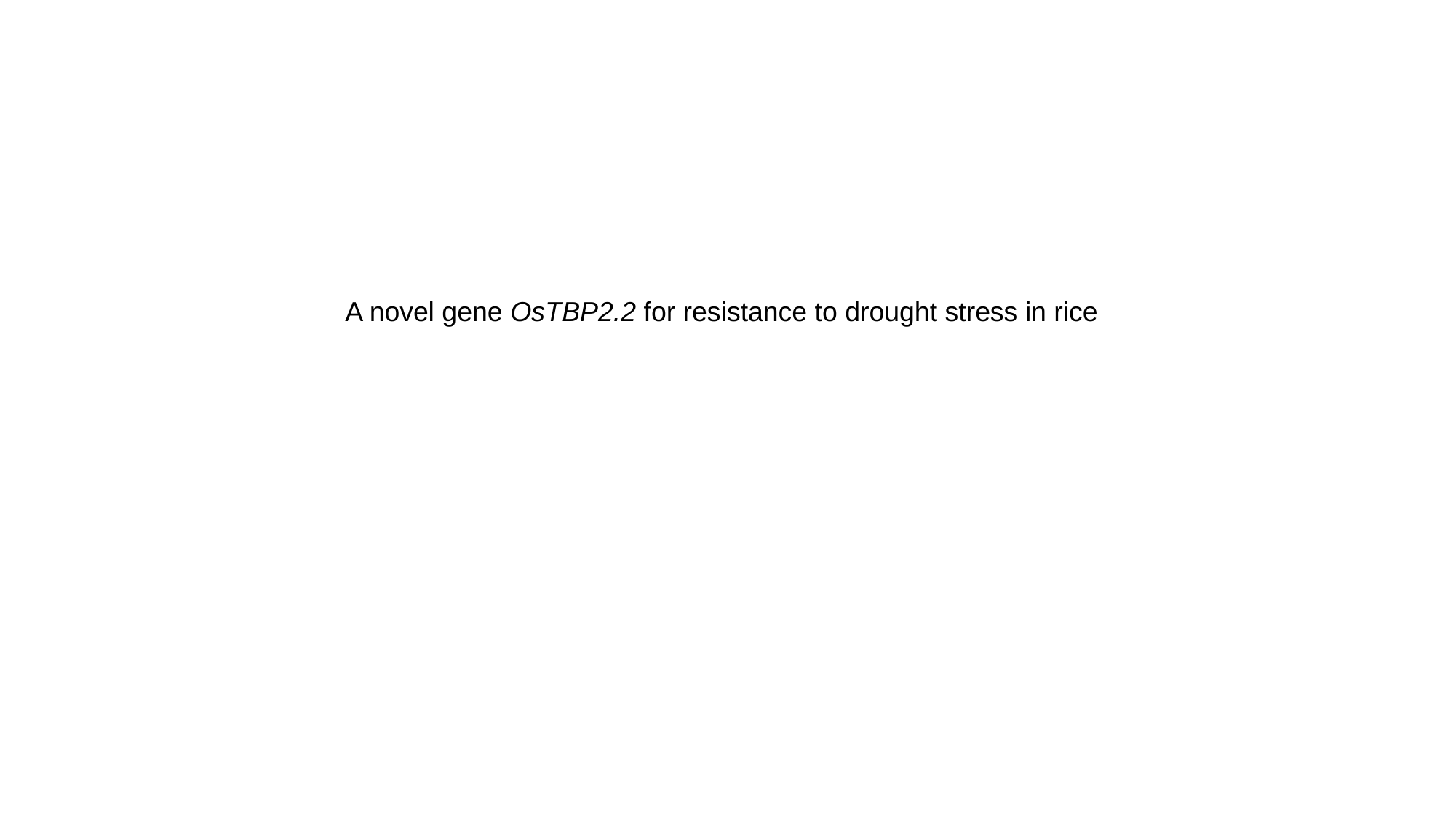

A novel gene OsTBP2.2 for resistance to drought stress in rice

## Slide 2
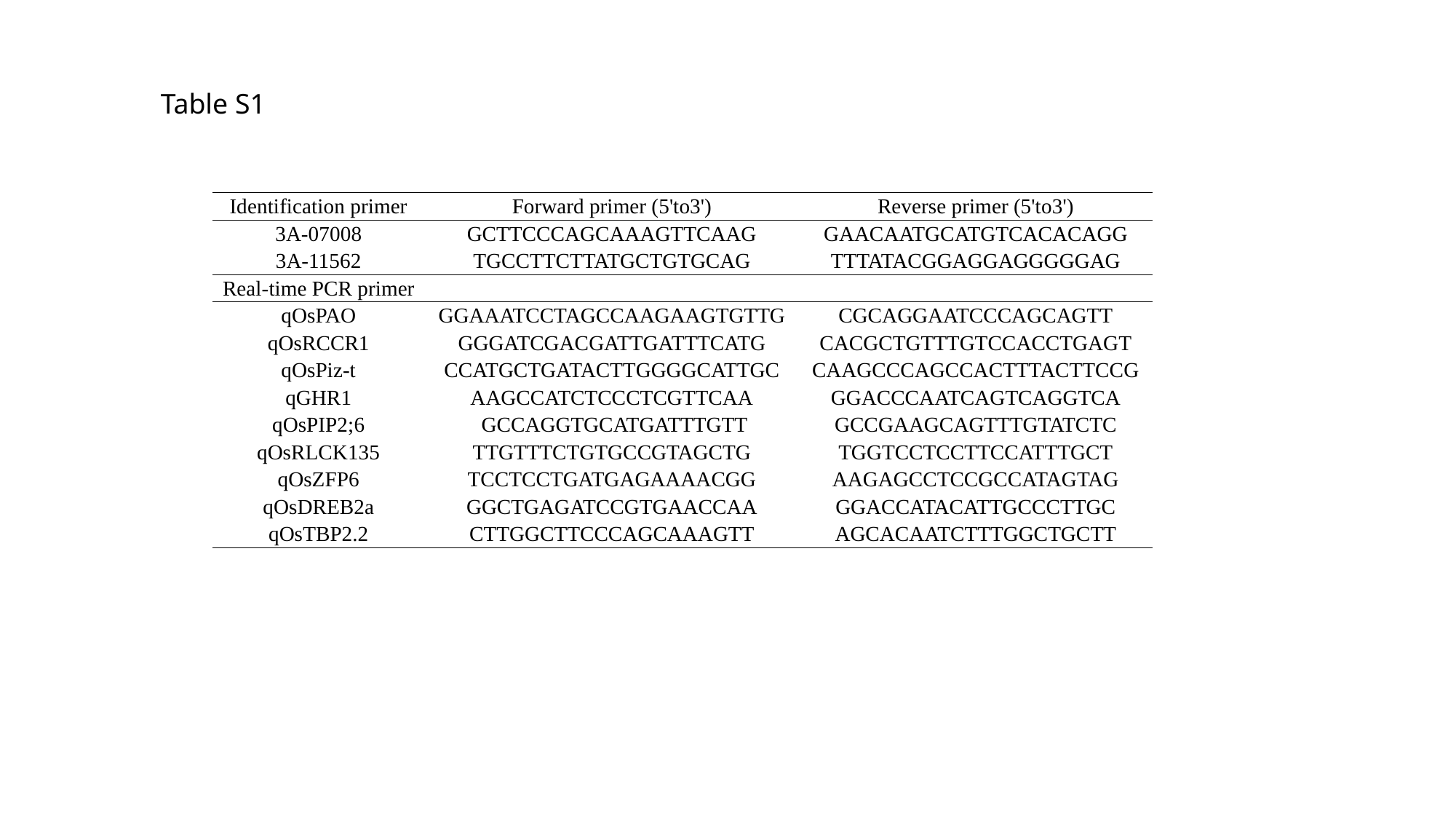

Table S1
| Identification primer | Forward primer (5'to3') | Reverse primer (5'to3') |
| --- | --- | --- |
| 3A-07008 | GCTTCCCAGCAAAGTTCAAG | GAACAATGCATGTCACACAGG |
| 3A-11562 | TGCCTTCTTATGCTGTGCAG | TTTATACGGAGGAGGGGGAG |
| Real-time PCR primer | | |
| qOsPAO | GGAAATCCTAGCCAAGAAGTGTTG | CGCAGGAATCCCAGCAGTT |
| qOsRCCR1 | GGGATCGACGATTGATTTCATG | CACGCTGTTTGTCCACCTGAGT |
| qOsPiz-t | CCATGCTGATACTTGGGGCATTGC | CAAGCCCAGCCACTTTACTTCCG |
| qGHR1 | AAGCCATCTCCCTCGTTCAA | GGACCCAATCAGTCAGGTCA |
| qOsPIP2;6 | GCCAGGTGCATGATTTGTT | GCCGAAGCAGTTTGTATCTC |
| qOsRLCK135 | TTGTTTCTGTGCCGTAGCTG | TGGTCCTCCTTCCATTTGCT |
| qOsZFP6 | TCCTCCTGATGAGAAAACGG | AAGAGCCTCCGCCATAGTAG |
| qOsDREB2a | GGCTGAGATCCGTGAACCAA | GGACCATACATTGCCCTTGC |
| qOsTBP2.2 | CTTGGCTTCCCAGCAAAGTT | AGCACAATCTTTGGCTGCTT |
